# Upregulated MicroRNA-92b Regulates the Differentiation and Proliferation of EpCAM-Positive Fetal Liver Cells by Targeting C/EBPß

**DOI:** 10.1371/journal.pone.0068004

**Published:** 2013-08-02

**Authors:** Nian-Song Qian, Wei-Hui Liu, Wen-Ping Lv, Xin Xiang, Ming Su, Vikram Raut, Yong-Liang Chen, Jia-Hong Dong

**Affiliations:** 1 Department of Hepatobiliary Surgery, PLA General Hospital, Beijing, China; 2 Department of Hepatobiliary Surgery, Hainan Branch of PLA General Hospital, Sanya, China; 3 Department of Hepatobiliary Surgery, General Hospital of Chengdu Military Region, Chengdu, China; 4 Department of Hepatobiliary Surgery, Graduate School of Medicine, Kyoto University, Kyoto, Japan; Xiangya Hospital of Central South University, China

## Abstract

microRNAs (miRNAs) are short noncoding RNAs that negatively regulate gene expression. Although recent evidences have been indicated that their aberrant expression may play an important role in cancer stem cells, the mechanism of their deregulation in neoplastic transformation of liver cancer stem cells (LCSCs) has not been explored. In our study, the HCC model was established in F344 rats by DEN induction. The EpCAM^+^ cells were sorted out from unfractionated fetal liver cells and liver cancer cells using the FACS analysis and miRNA expression profiles of two groups were screened through microarray platform. Gain-of-function studies were performed *in vitro* and *in vivo* to determine the role of miR-92b on proliferation and differentiation of the hepatic progenitors. In addition, luciferase reporter system and gene function analysis were used to predict miR-92b target. we found that miR-92b was highly downregulated in EpCAM^+^ fetal liver cells in expression profiling studies. RT-PCR analysis demonstrated reverse correlation between miR-92b expression and differentiation degree in human HCC samples. Overexpression of miR-92b in EpCAM^+^ fetal liver cells significantly increased proliferation and inhibited differentiation as well as *in vitro* and *in vivo* studies. Moreover, we verified that C/EBPß is a direct target of miR-92b and contributes to its effects on proliferation and differentiation. We conclude that aberrant expression of miR-92b can result in proliferation increase and differentiation arrest of hepatic progenitors by targeting C/EBPß.

## Introduction

Hepatocarcinogenesis is a complex process involving heterogeneous cellular and molecular variations. Recent cancer stem cell hypothesis supports the heterogeneous cellular origin of cancer from endogenous stem cells [1]. In some human HCC cell lines, a subset of liver cancer stem cells (LCSCs) have been identified and characterized by their self-renew capacity and high tumorigenicity [2]. These LCSCs, specifically express surface stem cell markers like CD90 [3], CD133 [4] and EpCAM [5]. According to the cancer stem cells hypothesis, cancer stem cells evolve by neoplastic transformation of normal somatic stem cells or progenitors, which may not be verified by only characterizing stem-like subpopulation from immortalized cancer cell lines without syngeneic normal tissue-specific stem cells as reference. Nevertheless, animal carcinogenesis models have proven this hypothesis; the rodent chemical hepatocarcinogenesis model has been now recognized as one of the typical cancer stem cell model [6]. Increasing experimental evidence suggests that EpCAM is the earliest marker expressed by the hepatic stem cells. Moreover, recent studies also indicate that EpCAM^+^ HCC cells are tumor-initiating cells with stem cell features [7]. So in this model both normal hepatic stem cells and LCSCs would be enriched by EpCAM, then the neoplastic transformation mechanism of LCSCs would be explored.

These cell lineages of multipotent stem cells are regulated by tissue-specific microRNAs (miRNAs). miRNAs are non-coding RNAs of 19 to 25 nucleotides in length that regulates the gene expression by inducing translational inhibition and cleavage of their target mRNAs through base pairing to partially or fully complementary sites [8]. Moreover, reports also indicate that dysregulation of miRNAs occurs frequently in a variety of carcinomas, including HCC [9]. The dual regulating effects of miRNAs in both carcinogenesis and differentiation of stem cells strongly suggest that miRNAs may be involved in the neoplastic transformation of normal stem cells into cancer stem cells. To explore the cellular origin and its molecular signature of LCSCs, an available and novel strategy is to determine changes in the expression profiles of specific miRNAs and their target messenger RNAs (mRNA) between normal hepatic stem cells and LCSCs during hepatocarcinogenesis in an animal model.

Our survey detected increases of miR-92b during hepatocarcinogenesis. Furthermore, gain-of-function studies were performed *in vitro* and *in vivo* to determine the role of miR-92b in the hepatic progenitors. This study clarifies that overexpression of miR-92b would result in proliferation increase and differentiation arrest of hepatic progenitors by targeting CCAAT/enhancer binding protein beta (C/EBPß) gene.

## Materials and Methods

### Establishment of animal model and cell culture

Chemical hepatocarcisnogenesis F344 rat model was established according to the previous report [10]. Thirty male Fisher 344 rats (from the National Rodent Laboratory Animal Resource, Shanghai, China) were randomly divided into control and trial groups. Rats in the trial group were treated with 0.05% DEN (Sigma Co, USA) in their drinking water for 6 weeks and were then changed to normal drinking water, whereas rats in the control group were given a normal diet. Three rats from each group were sacrificed under anesthesia at 2, 6, 10, 14 and 18 weeks after DEN induction. Both HCC nodules from the trial group and normal livers from the control group were collected. The remaining tissues were used directly in the experiments detailed below. All animal experiments were performed in accordance with animal study protocols and approved by the Research Animal Care and Use Committee at the PLA General Hospital (Beijing, China). Primary fetal liver cells were obtained from day 14 rat embryo and cancer cells were isolated from HCC nodules. The hepatic maturation were induced by HGF(20 ng/ml) [11]. EpCAM+ fetal liver cells or liver cancer cells were sorted by using a fluorescent activated cell sorting (FACS) [12].

### Clinical samples and data

The study was approved by the Institutional Clinical Ethics Committee of PLA General Hospital (Beijing, China). One hundred and fifty-two patients (124 males and 28 females) who underwent radical resection between 2008 and 2009 at PLA General Hospital were enrolled for this study. The study was approved by the Institutional Clinical Ethics Committee of PLA General Hospital (Beijing, China). All the participants provide their written informed consent to participate in this study. Resected liver specimens were collected after an informed consent. Gene expression analysis was conducted on a primary HCC and adjacent non-cancerous hepatic tissues using frozen section. If miR-92b expression in tumor tissues was 2 times higher than in the nontumoral liver tissues, it was considered as a “high” result. On the contrary, the “low” was that the expression level in tumor tissues was 50% lower than that in the nontumoral liver tissues [13], [14].

### Microarray analysis

The miRCURY Hy3/Hy5 labeling kits (Exiqon, Vedbaek, Denmark) were used to label purified miRNA with Hy3TM or Hy5TM fluorescent dye. Labeled samples were hybridized on the miRCURY LNA (locked nucleic acid) Array (version 11.0, Exiqon, Vedbaek, Denmark) and run in quadruplicate. Background subtraction and normalization were performed. The miRNAs with at least 1.5 fold differences in expression levels between two groups were selected for further analysis.

### Expression vector constructs and transfection

Pre-miR miRNA precursors (pEZX-92b) were purchased from GeneCopoeia (GeneCopoeia, Rockville, USA). Target gene's expression vectors with 3′UTR or mutant 3′UTR were constructed by using eukaryotic expression vector pcDNA3.1 (+). The inserted full-length target genes cDNA with 3′UTR were purchased from OriGene (OriGene, Rockville, USA). The expression constructs with mutant 3′UTRs were generated by using a QuickChange II Site-Directed Mutagenesis Kit (Stratagene, Cedar Creek, TX). The primers were listed together with the nucleotide sequences of all the PCR cloning primers (Table 1).

**Table 1:** Association of mirR-92b with clinicopathological characteristics of HCC patients (n = 152).

Parameters	Variable	Mir-92b		
		low	High	*P*
Age	≤ 50	28% (22)	39% (29)	
	>50	72% (55)	61% (46)	0.189
Sex	Male	78% (60)	85% (64)	
	Female	22% (17)	15% (11)	0.240
HBS-Ag	Positive	88% (66)	83% (62)	
	Negative	12% (11)	17% (13)	0.608
HCV-Ab	Positive	14% (11)	8% (6)	
	Negative	86% (66)	92% (69)	0.220
Cirrhosis	Positive	71% (55)	75% (56)	
	Negative	29% (22)	25% (19)	0.654
Tumor size	≥ 5cm	16% (12)	27% (20)	
	<5cm	84% (65)	73% (55)	0.095
Histological differentiation	Well-differentiated	38% (29)	22% (17)	
	Moderately-differentiated	35% (27)	39%(29)	
	Poor-differentiated	27% (21)	39% (29)	0.042

HBs-Ag, hepatitis B virus antigen; HCV-Ab, hepatitis C virus antibody.

Ectopic miR-92b overexpressed EpCAM^+^ fetal liver cells were established as follows: Firstly, lentiviral infection was performed on the primary cultures of fetal liver cells. Then the stable transfectants were selected and expanded by using hygromycin or neomycin. Finally, the miR-92b overexpressed EpCAM^+^ cells could be enriched by performing FACS to the transfectants for further study.

### Prediction and identification of miR-92b targets

Rat target genes potentially regulated by miR-92b were searched from the website (http://www.microrna.org). Its target predictions are based on a development of the miRanda algorithm and mirSVR scoring [15]. mirSVR downregulation scores are calibrated to correlate linearly with the extent of downregulation and therefore, enable accurate scoring of genes with multiple target sites by simple addition of the individual target scores. Furthermore, the scores can be interpreted as an empirical probability of downregulation, which provides a meaningful guide for selecting a score cutoff [16]. In this study, those miRNA/target duplexes with mirSVR scores less than −0.10 were selected as candidate miR-92b target sites.

Gene expression analysis comparing miR-92b overexpressed EpCAM^+^ fetal liver cells and control cells was performed with a custom cDNA microarray containing potential target genes of miR-92b. We submitted the samples to KangChen-Biotech (Shanghai, China) for array hybridization on the customized cDNA array (SABiosciences, Frederick, USA).

### 3′UTR reporter assay

The 3′UTR fragments of the target genes were subcloned into the pGL3-control luciferase reporter vectors (Promega, Madison, USA). 3′UTR reporter assay was performed with using EpCAM^+^ fetal liver cell. Firstly, EpCAM^+^ fetal liver cell infected with pEZX-92b lentivirus, and then they were seeded in 6-well plates and cotransfected with 0.5 g of the respective pGL3-3′UTR or pGL3-control constructs and 0.05 g of the pRL-TK vector using the FuGENE® HD Transfection Reagent (Promega, Madison, USA). After transfection, the cells were split into 24-well plates and harvested for luciferase assays 48 hours using the Dual-Luciferase Reporter Assay System kit (Promega, Madison, USA) according to the manufacturer's protocol.

### *In vitro* & *in vivo* differentiation of EpCAM^+^ fetal liver cells assay

*In vitro* differentiation of transfected EpCAM+ fetal liver cells were performed according to previous reports [17], [18]. Induced differentiation was completed by 15 days of culture. After induction, morphologic characteristics of the cells were observed by using phase contrast microscopy and transmission electron microscopy. Characterized gene expression analyses of hepatocyte also were performed by using real-time RT-PCR, western blot and immunohistochemistry, which include albumin (ALB) and alphafetoprotein (AFP). Metabolic capacity during progress of induction, including urea synthesization, ALB secretion and glycogen storage, were used to evaluate the differentiation degrees of hepatocyte-like cells. Capacity of urea synthesis was measured with a colorimetric assay kit (Sigma, St.Louis, USA). Glycogen store was measured using periodic acid schiff (PAS) staining.

*In vivo* differentiation of transfected EpCAM^+^ fetal liver cells were performed by transplantation into the F344 rat livers injured by carbon tetrachloride and two/thirds partial hepatectomy (PH). Control cells and mir-92b overexpressed cells were independently washed with PBS in the dark and resuspended in 2 ml staining solution to label the cell membrane with red fluorescence or GFP, respectively. Serum-containing media was added to the staining solution to terminate the staining 5 min later. Stained cells were washed three times with PBS and suspended in 0.5 ml PBS for transplantation. Finally, the prepared cells (5×10^5^ cells per rats) were injected respectively through portal vein after PH. The rats were sacrificed 4 weeks later for histopathological evaluation [11].

### Cell cycle analysis

For cell cycle analysis, transfected cells in the log phase of growth were obtained by trypsinization and pooled with the floating cells and centrifuged at 1000 rpm for 5 min. Propidium iodide (0.05 mg/ml, Sigma, St.Louis, USA) and RNAseA (0.1 mg/ml, Sigma, St.Louis, USA) were added to the cells and samples were analyzed 30 min after staining with the use of flow cytometry-BD FACSCalibur (BD) and Cell-Quest software.

### Real-time RT-PCR

Real-time RT-PCR was performed to analyze miRNA and mRNA expression levels. Real-time PCR was per-formed using SYBR PrimeScript RT-PCR Kit in the Light Cycler System (Roche Diagnostics, Lewes, UK). The primer sequences used for PCR were listed in Table 1. Amplification was performed with the following cycles: 95°C for 30 s, followed by 40 cycles of denaturing at 95°C for 5 s and annealing at 60°C for 20 s. All of the reactions were performed in triplicate. Data analysis was performed using the 2^−ΔΔCt^ method [19]. β-actin was used as a reference gene.

### Western Blot

Cellular proteins were extracted and separated in SDS-PAGE gels, and western blot analyses were performed according to standard procedures. Western blotting of ß-actin on the same membrane was used as a loading control. The antibodies used were anti- EpCAM, anti-C/EPBß, anti-Foxg1, anti-Gaat2 and anti-ß-actin (Santa Cruz Biotechnology, CA, USA).

### Statistical Analysis

The results were expressed as mean ± SD, and were analyzed with SPSS 17.0 for Windows. The statistical significance of the observed differences between groups was analyzed by Student's *t* test or the χ^2^ test. The relationships between the miR-92b and other mRNA expressions were analyzed by correlation coefficients and linear regression analysis. *P*<0.05 was considered to be statistically significant.^ *^ *P*<0.05; ^**^ *P*<0.01.

## Results

### miR-92b was downregulated in EpCAM^+^ fetal liver cells

**Figure 1:**
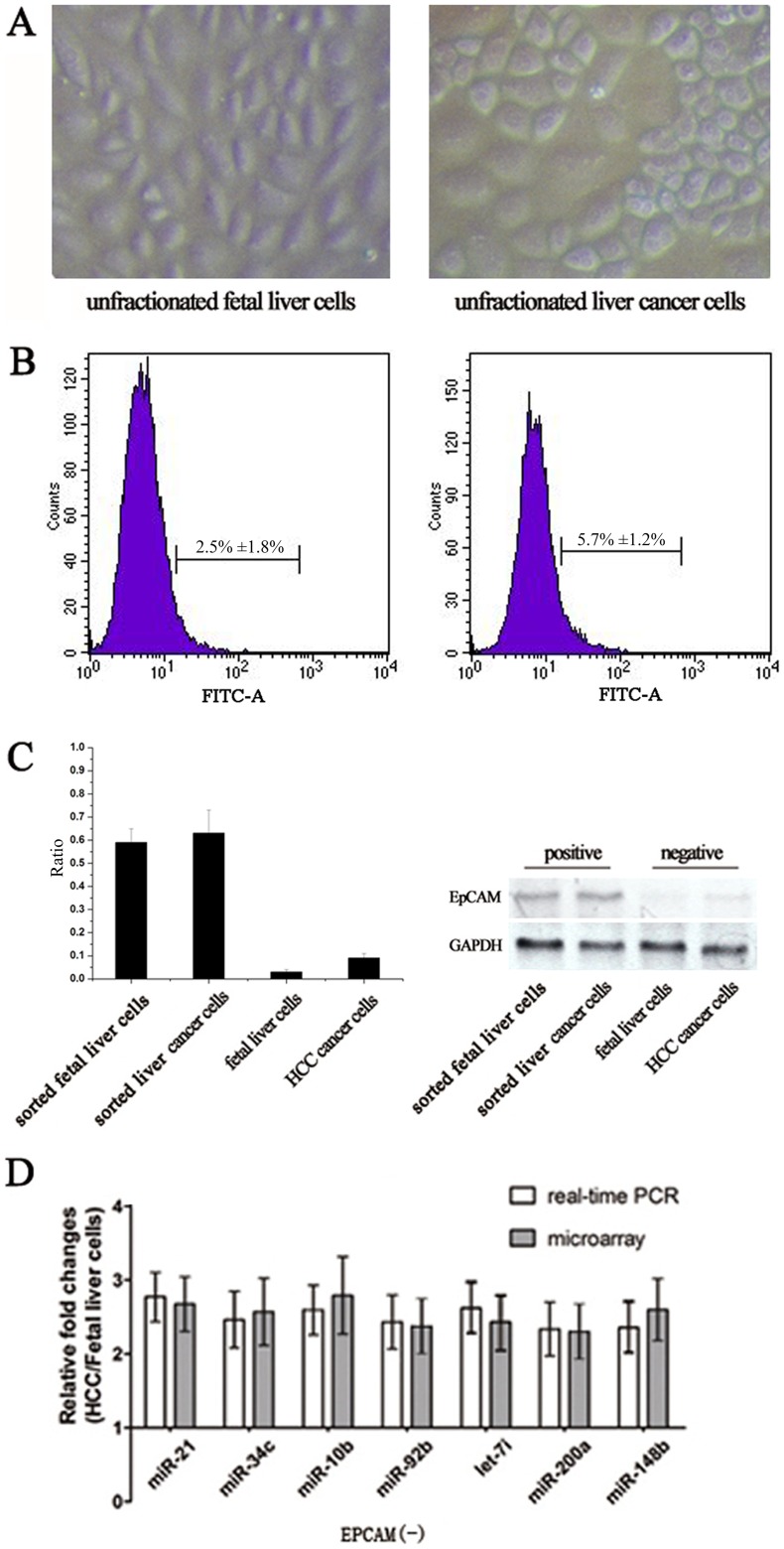
Primary EpCAM^+^ cells enrichment and miRNAs microarray analysis. (A) phase-contrast microscopic observation showed that unfractionated primary fetal liver cells and liver cancer cells had similar appearance; (B) histograms representing the isolated EpCAM^+^ cells derived from the fetal liver cells and liver cancer cells respectively by using FACS analysis; (C) Real-time RT-PCR and Western Blot analysis showed that none of the EpCAM^+^ cells were in the EpCAM^−^ fraction after enrichment by using FACS sorting; (D) Real-time RT-PCR analysis validated fold changes of partial miRNAs dysregulated in microarray analysis between EpCAM^−^ groups.

Embryonic 14-day F344 rat fetal liver were used as the cellular source of normal hepatic progenitors, and neoplastic nodules of HCC in F344 rats induced with DEN as the cellular source of LCSCs. Epithelial-like cell clusters were studied under phase contrast microscopy after inoculation (Fig. 1A). The EpCAM^+^ cells were sorted out from unfractionated fetal liver cells and liver cancer cells using FACS analysis; 2.5%±1.8% of the fetal liver cells and 5.7%±1.2% of the liver cancer cells were expressing the EpCAM receptors (Fig. 1B). The expression of EpCAM in these sorted cells was confirmed by real-time RT-PCR and western Blot (Fig. 1C). Although there is no widely accepted marker for liver stem cells, EpCAM is one of the most widely used markers [7], [20], [21]. To further confirm the results of EpCAM investigation, AFP expression level on the sorted EpCAM +/− cells were detected. The related results were showed in Fig. S1.

Our previous report analyzed miRNAs of the two groups using microarray, to identify critical miRNAs involved in a neoplastic transformation of LCSCs during the hepatocarcinogenesis. The miRNAs microarray analysis showed deregulation of 78 miRNAs in EpCAM^+^ liver cancer cells as compared with EpCAM^+^ fetal liver cells (*P*<0.05). Among these miRNAs, 68 were up-regulated and 10 were down-regulated. miR-92b was the most up-regulated miRNA in this profiling study, with 3.56±1.02 fold changes after normalization (q = 0.000, FDR = 0.036). Similarly, the expression of oncogenic miRNAs like miR-21, miR-10b, let-7i, miR-34c, were increased more than 2 fold in EpCAM^+^ liver cancer cells; whereas miR-125b, miR-200a, miR-148b were most down-regulated. Our previous report also revealed that the relative expression levels of miR-92b, miR-21, miR-34c, miR-10b, and let-7i in EpCAM^+^ liver cancer cells compared to fetal liver cells were increased (*P*<0.05). In addition, the miR-200a and miR-148b were significantly underexpressed in EpCAM^+^ liver cancer cells compared to fetal liver cells (*P*<0.05) [22]. However, the expression levels of these miRNAs were not significantly changed between EpCAM^−^ liver cancer cells and EpCAM^−^ fetal liver cells (*P*>0.05). The expression patterns of these miRNAs in RT-PCR were similar to our findings in microarray profiling study (Fig. 1D).

### miR-92b is upregulated in human HCC and associated with histological differentiation

**Figure 2:**
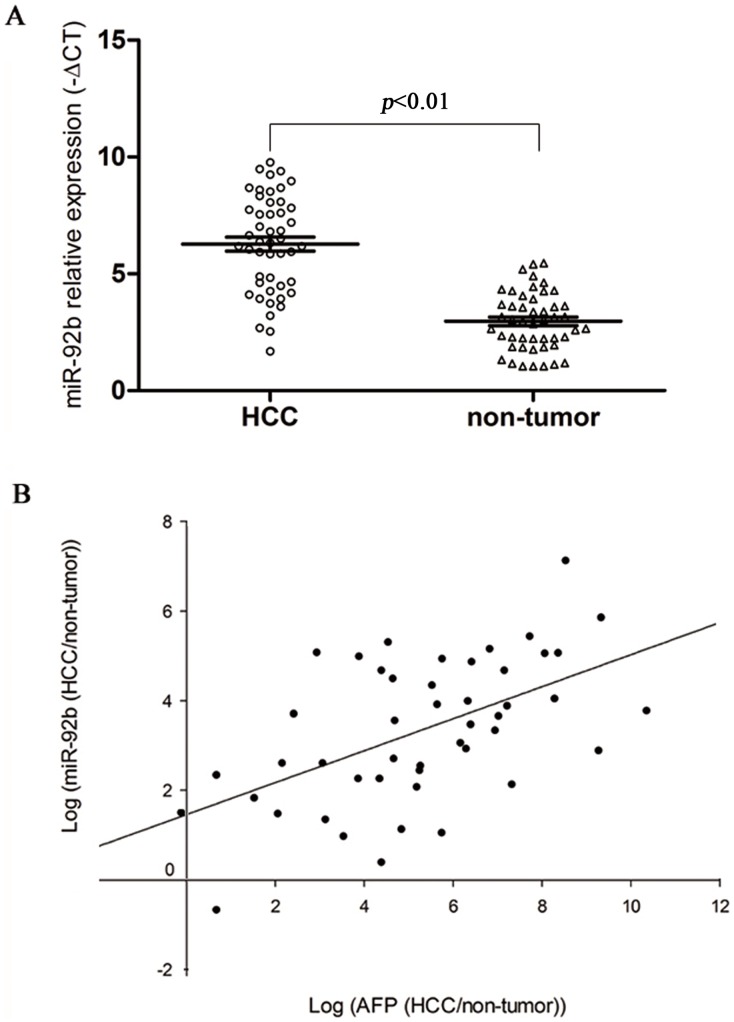
miR-92b expression in clinical HCC samples. (A) miR-92b was differentially expressed between HCC and the corresponding nontumoral liver tissues. (B) miR-92b expression demonstrated a significant positive correlation with AFP mRNA (*P*<0.05).

miR-92b was the most upregulated miRNA in our profiling studies, and we selected it for further analysis. Among the 152 HCC clinical samples, relative expression of miR-92b in HCC was significantly higher compared to nontumoral liver tissues (6.25±2.08 *vs* 2.96±1.23, *P*<0.01. Fig. 2A). Moreover, expression of miR-92b was positively associated with AFP mRNA (r = 0.554, *P*<0.05, Fig. 2B). Next, the expression pattern of miR-92b was correlated with clinicopathological parameters of HCC. The relative expression of miR-92b was significantly associated with a degree of differentiation (*P*<0.05, Table 2).

**Table 2:** Primers.

Name	RT primer (5′–3′)	PCR Forward primer (5′–3′)	TM(°C)
EpCAM	CGCAGCTCAGGAAGAATGTG	TGAAGTACACTGGCATTGACG	60
CEBPB	AACCAACCGCACATGCAGAT	GGCAGAGGGAGAAGCAGAGAGT	60
has-miR-21	GTCGTATCCAGTGCAGGGTCCGAGGTATTCGCACTGGATACGACTCAACA	CGCGCTAGCTTATCAGACTGA	60
hsa-miR-34c-3p	GTCGTATCCAGTGCAGGGTCCGAGGTATTCGCACTGGATACGACCCTGGC	GGTGGAATCACTAACCACACG	60
has-miR-10b	GTCGTATCCAGTGCAGGGTCCGAGGTATTCGCACTGGATACGACCACAAA	CATGGTACCCTGTAGAACCGAA	60
has-let7i	GTCGTATCCAGTGCAGGGTCCGAGGTATTCGCACTGGATACGACAGCAAG	TAGTACTGCGCAAGCTACTGC	60
has-mir-200a	GTCGTATCCAGTGCAGGGTCCGAGGTATTCGCACTGGATACGACTCCAGC	GAGTGCATCTTACCGGACAGT	60
has-miR-148b	GTCGTATCCAGTGCAGGGTCCGAGGTATTCGCACTGGATACGACGCCTGA	GGCGCAAGTTCTGTTATACAC	60
miR-92b	UUGCACUUGUCCCGGCCUG	TATTGCACTCGTCCCGGCCTCC	60
AFP	AAATGCGTTTCTCGTTGC	CAGCCTCAAGTTGTTCCTCT	60
ALB	T GCTTGAATGTGCTGATGACAGGG	AAGGCAAGTCAGCAGGCATCTCATC	60
GAPDH	GTCAACGGATTTGGTCTGTATT	AGTCTTCTGGGTGGCAGTGAT	60
General primer	PCR Reverse Primer: GTGCAGGGTCCGAGGT		60

### miR-92b regulates cell proliferation and differentiation of EpCAM^+^ fetal liver cells

**Figure 3:**
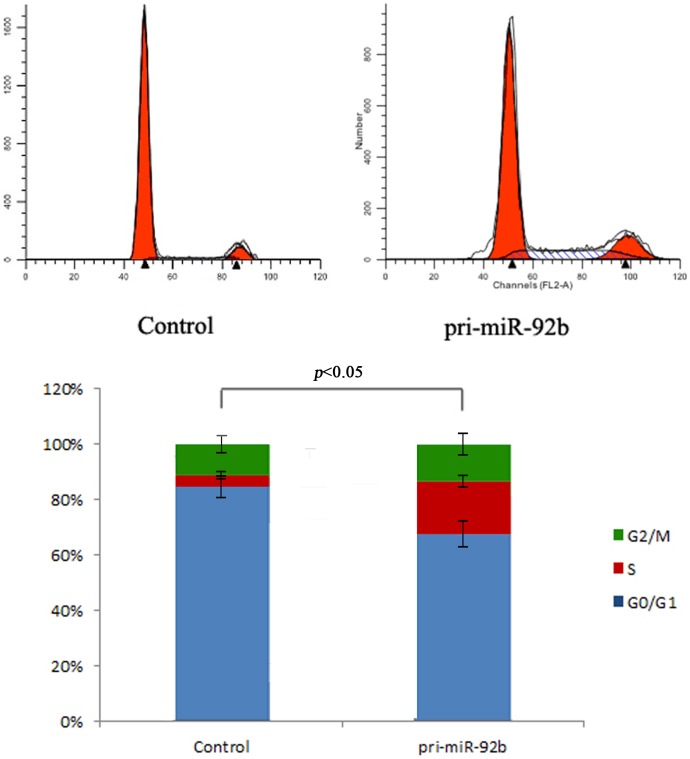
miR-92b regulated the proliferation of EpCAM^+^ fetal liver cells. Cell cycle analysis showed that overexpression of miR-92b in EpCAM^+^ fetal liver cells would result in the decrease in G_1_ phase and significantly increase in S and G_2_/M phase.

A series of biological function assays were performed on miR-92b overexpressed group or control group to explore the effect of miR-92b on primary cultures of hepatic progenitors. Firstly, we assessed the effect of miR-92b on modulation and proliferation of hepatic progenitors (Fig. 3). The percentage of miR-92b overexpressed and control groups in the G_0_/G_1_ phase respectively were 67.2%±2.2% and 84.3%±3.1% (*P*<0.05). S phase fractions in two groups of the cells were 19.3%±2.1% and 2.3%±1.2% (*P*<0.05), respectively. In addition, overexpression of miR-92b in EpCAM+ fetal liver cells would result in the significantly G2/M phase (13.5%±2.4% vs 11.4%±2.1%, *P*<0.05).

**Figure 4:**
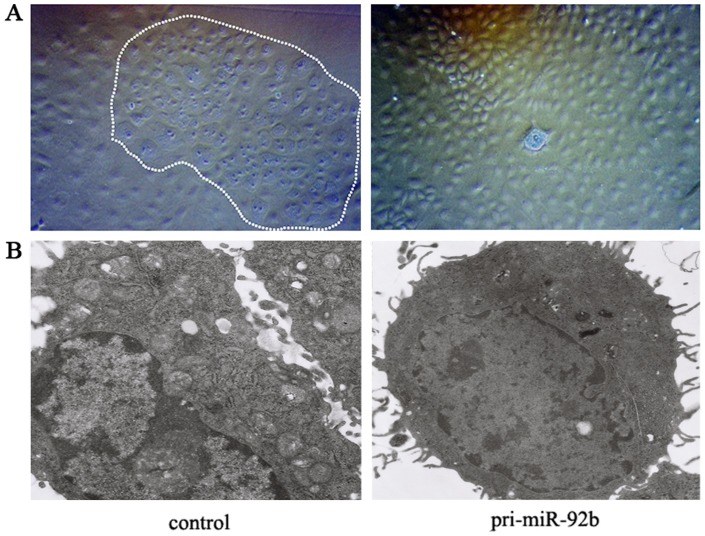
Effects of miR-92b on the hepatic differentiation and maturation of EpCAM^+^ fetal liver cells. (A) microscopic observation of miR-92b overexpressed and control groups after hepatocyte differentiation induction by using phase-contrast microscope. The control image was showing the mature degree and apoptosis of control cells compared to the mir-92b overexpressed cells, especially the cells in the white lines. However, most of the miR-92b overexpressed cells were similar to immature cells and still at the intermediate stage from hepatic progenitor to mature hepatocyte. (B) electron microscopic appearance of miR-92b overexpressed and control groups after induction. The ultrastructure was more preserved in control cells comparing to the miR-92b overexpressed cells. 100×(A), 8000×(B).

Further, we investigated the influence of miR-92b on the hepatic differentiation and maturation of EpCAM^+^ fetal liver cells. *In vitro* differentiation assay revealed the significant difference in the morphologic features of EpCAM^+^ cells between two groups, which were examined on both light and electron microscopy. Phase-contrast microscopy revealed the miR-92b overexpressed cells were similar to immature cells, at intermediate stage from hepatic progenitor to mature hepatocyte. These miR-92b overexpressed immature cells were small, mononuclear, round or oval-shaped and usually arranged in cobblestone-like appearance. Moreover, electron microscopic analysis showed that the ultrastructure of mature hepatocytes was preserved in cultured control cells. The well-developed rough endoplasmic reticulum, mitochondria, microvilli, glycogen granules, marginal chromatin and small nuclear/cytoplasm ratio, even biliary canaliculi structures were also observed in these cells. On other hands, the large, round, hyperchromatic unclei, poor number of cellular organelles, nonpolarity, with the increased nuclear/cytoplasm ratio were observed in the miR-92b overexpressed cells as a phenotype in the liver progenitor-like cells (Fig. 4).

**Figure 5:**
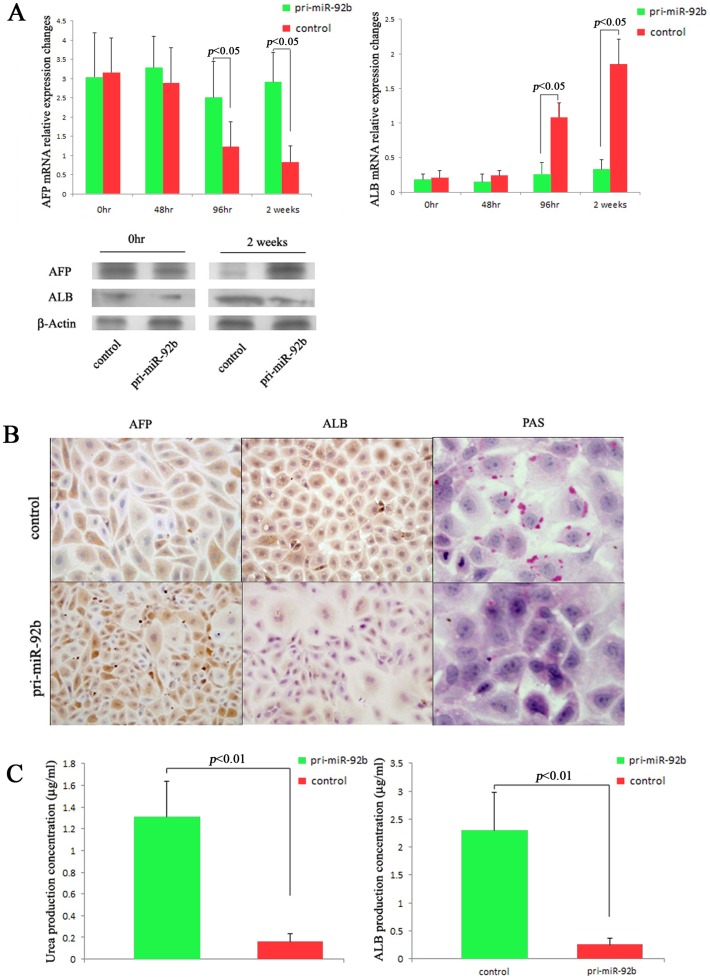
Effects of miR-92b on the differentiation arrest of EpCAM^+^ fetal liver cells. (A) AFP and ALB mRNA expressions in miR-92b overexpressed and control groups after induction. The western blotting images showed the most significant difference of APF and ALB expressions between two groups 2 weeks after induction. (B) immunohistochemical staining of AFP, ALB and PAS in miR-92b overexpressed and control groups 2 weeks after induction; (C) detection and analysis of urea and ALB production secreted into the medium in miR-92b overexpressed and control groups. 200×(B).

Further, we studied the hepatocyte-specific biochemical functions to prove the hepatocyte differentiation arrest caused by overexpression of miR-92b. There was no significant difference between these two groups according to AFP and ALB mRNA expressions on 0 h or 48 h after differentiation induction. However, AFP mRNA level in the miR-92b overexpressed group were significantly increased in comparison with the control group on 96 h and 2w after differentiation induction (*P*<0.05 and <0.05, respectively). Compared with the control group, the miR-92b overexpressed group had significantly decreased ALB mRNA levels (*P*<0.05). We also checked the protein expressions of AFP and ALB on 2w after differentiation induction (Fig. 5A). The results were similar to the above report. In addition, control group was positive for PAS staining indicating the glycogen storages, while the differentiated miR-92b overexpressed group was negative for PAS staining (Fig. 5B). Then, we studied the ability of these two groups to secret ALB and synthesize urea and compared during induced differentiation. ALB and urea secretion had been increased from day 7 and 9 respectively reaching maximal values on day 14 in the medium of control group, which were dramatically higher than that in the medium of miR-92b overexpressed group (*P*<0.05, Fig. 5C).

**Figure 6:**
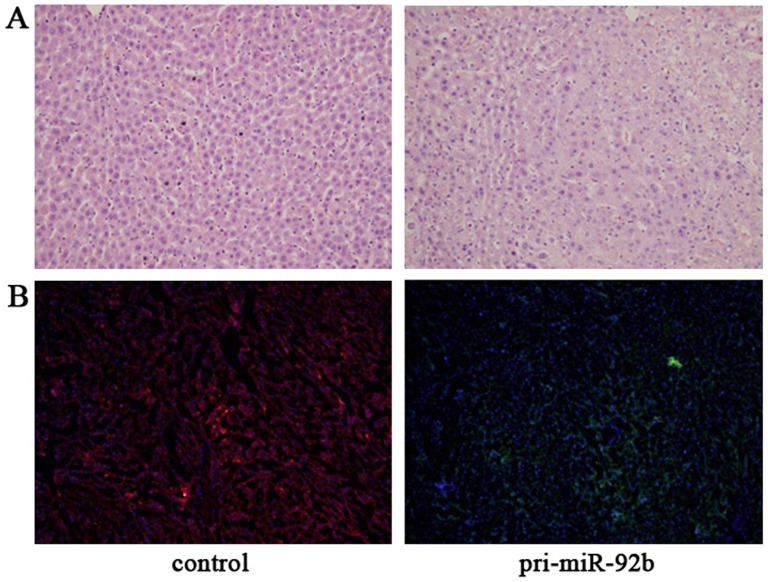
*In vivo* differentiation assay of the effect of miR-92b overexpression on liver repopulation capacity of EpCAM^+^ fetal liver cells. (A) Histological examination verified the significant differences in the *in vivo* hepatic differentiation between two groups; (B) Tracing the differentiation of miR-92b overexpressed and control groups in the liver by using fluorescent microscope. Large clusters of red fluorescence-positive cells with hepatocyte morphology were observed in the control group, and only small satellite clusters of GFP positive cells with the small oval cell morphology could be observed in the miR-92b overexpressed group. 200×(A and B).

The effect of miR-92b overexpression on repopulation capacity of EpCAM^+^ fetal liver cells was studied with *in vivo* differentiation assay. The EpCAM^+^ transfected fetal liver cells or control cells were transplanted into two groups of F344 rat livers, injured by carbon tetrachloride and two/thirds PH. These livers were studied after 4 weeks of cell injection. Histopathogical examination showed that the degree of repair was better in livers of the rats injected with control group than the rats injected with miR-92b overexpressed group (Fig. 6A). Repopulation of injured liver by transfected fetal liver cells was then traced using a fluorescence microscope. Large clusters of red fluorescence-positive cells with hepatocyte morphology were observed in the area of hepatic lobules in the tissue sections transplanted with control group. Whereas, only small satellite clusters of GFP positive cells with the small oval cell morphology embedded in the region of the hepatic lobule closed to portal area could be observed in the sections transplanted with miR-92b overexpressed group (Fig. 6B). In addition, miR-92b overexpressed group and control group were orthotopically transplanted into the livers of nude mice. The livers transplanted with miR-92b overexpressed cells had a typical variegated appearance with extensive lesion. Under microscopic observation, the liver section transplanted with miR-92b overexpressed cells showed that portal area were filled with plenty of small oval immature cells, these cells extensively invaded into the adjacent lobule and destroy associated structure of lobules. It suggests that single overexpression of miR-92b in hepatic stem cells would result in abnormal proliferation (data not shown).

### C/EBPß is a direct functional target of miR-92b

**Figure 7:**
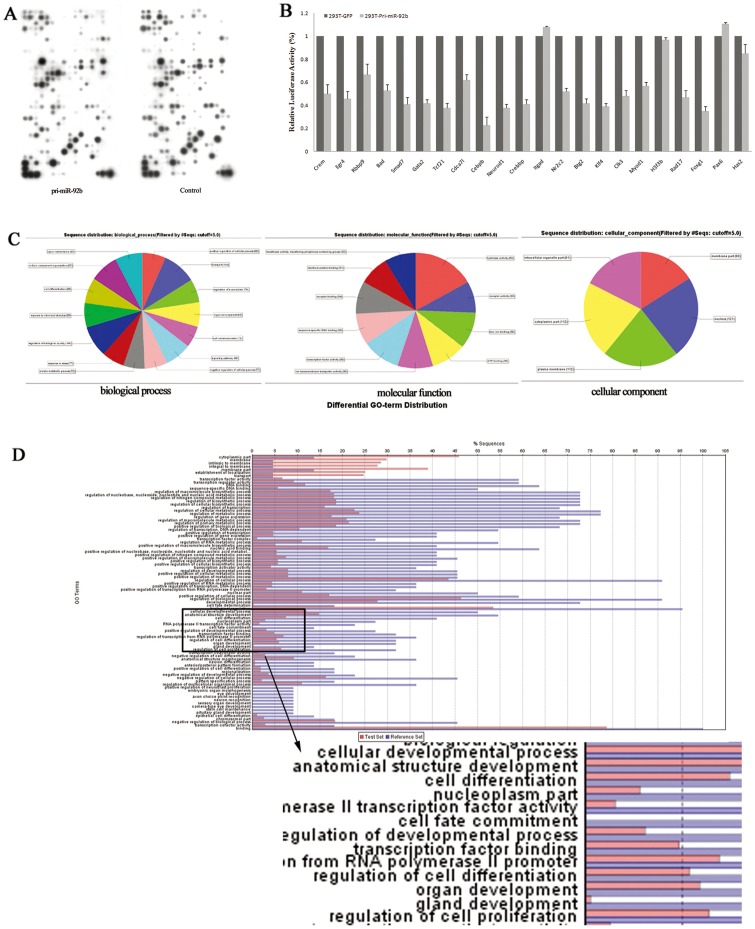
Predicting functional target genes of miR-92b. (A) *In vivo* screening potential targets of miR-92b in EpCAM^+^ fetal liver cells by using customized microarray analysis. The data showed that 7.96% (34/427) of predicted target genes in miR-92b overexpressed EpCAM^+^ cells were expressed lower than that in the counterpart. And 22 genes of which that relative fold changes less than 0.5 were considered as potential function targets of miR-92b; (B) Overall, 18 of the 22 3′UTR constructs demonstrated a significant reduction in luciferase activity for 293T cells overexpressing miR-92b (293T-pri-miR-92b), especially C/EBPß; (C) Potential targets could be classified in three aspects: biological process, molecular function and cellular component by using gene function analysis software; (D) Gene ontology analysis of the predicted target genes was performed with Blast2go software. The functions of many target genes were grouped in the categories of cell differentiation, transcription factor, organ development and regulation of cell proliferation, for choosing the genes involved in differentiation that specially mediated by mir-92b.

According to the mirSVR scores of miRNA/target duplexes are less than 0.1, 3′UTRs on the total of 427 gens were predicted as candidate target sites of miR-92b. Then customized microarray analysis was performed on miR-92b overexpressed and control groups enriched by FACS. Results of microarray analysis showed that 7.96% (34/427) of predicted target genes in miR-92b overexpressed EpCAM^+^ cells were expressed lower than in counterpart. Within these genes, 22 genes which expression was decreased by <0.5-fold in miR-92b overexpressed EpCAM^+^ cells were thought as the most probable target genes of miR-92b (Fig. 7A). The luciferase reporter assay was performed access whether the repressions of these genes are mediated by miR-92b specifically binding predicted sites in their 3′UTRs. As shown in Fig. 7B, a significant reduction in luciferase activity (23%–67%) was detected with 18 of the 22 cloned reporter constructs.

Next, we studied the function of these potential target genes. As Fig. 7C shown, candidate target genes could be classified in three aspects: biological process, molecular function and cellular component. Genes classified as having the biological process involved in “epithelial cell differentiation,” and “cell fate determination”, or having the molecular function involved in “transcription factor activity” were identified. The overlap between these biological processes and molecular functions pinpointed three genes, C/EBPß, Gata2 (GATA binding protein 2), Foxg1 (forkhead box G1) (Fig. 7D).

**Figure 8:**
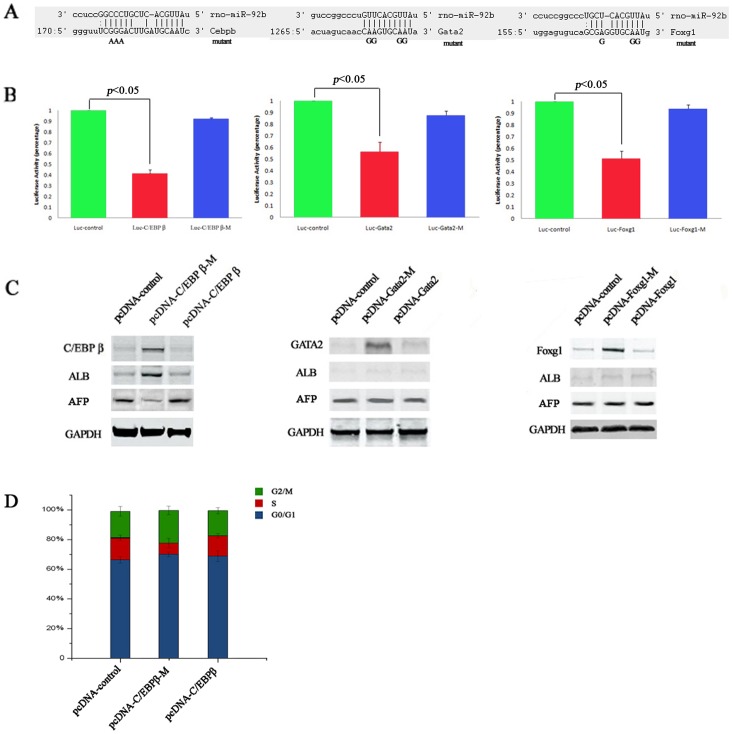
C/EBPß is a direct functional target of miR-92b. (A) Diagram depicting the 3′UTR reporter assay. The 3′UTR of the C/EBPß, Gata2 and Foxg1 genes contain putative target sites with the position of seed sequences as indicated; (B) Fetal liver cells transfected with pri-miR-92b and a luciferanse reporter containing a fragment of the 3′UTR harboring either the miR-92b binding site (Luc-C/EBPß, Luc-Gata2, Luc-Foxg1) or a mutant (Luc-C/EBPß-M, Luc-Gata2-M, Luc-Foxg1-M). The assays showed that luciferase activities in the Luc-C/EBPß, Luc-Gata2, Luc-Foxg1 groups were significantly decreased compared to the luciferase activities of the mutant and control groups; (C) When restoring the C/EBPß expression in miR-92b overexpressed fetal liver cells by transfecting pcDNA-C/EBPß-M constructs, increase of ALB production in miR-92b overexpressed fetal liver cells could be observed, while there are no significant changes about ALB production in mir-92b overexpressed fetal liver cells transfected with pcDNA-C/EBPß, pcDNA-Foxg1, pcDNA-Foxg1-M, pcDNA-Gata2 and pcDNA-Gata2-M, respectively. (D) Cell cycle analysis showed the rescue effects of C/EBPß in miR92b overexpressed fetal liver cells. The significantly increase in G1 phase and decrease in S and G2/M phase could be observed when restoring the C/EBPß expression in miR-92b overexpressed fetal liver cells by transfecting pcDNA-C/EBPß-M.

To assess the direct functional target of miR-92b involved in differentiation arrest of fetal liver cells, we initially investigated the binding sites of miR-92b in 3′UTR of three pinpointed target genes (C/EBPß, Gata2, Foxg1). Luciferase reporter constructs with 3′UTR or mutant 3′UTR of targets and control construct were transfected into miR-92b overexpressed fetal liver cells. This assays showed that the luciferase activity in the cells transfected with Luc-C/EBPß, Luc-Gata2 and Luc-Foxg1 were all significantly decreased compared to in the cells transfected with PGL3-control (*P* = 0.016, 0.047, 0.036, respectively). Moreover, the most reduction of luciferase activity was observed in cells transfected with Luc-C/EBPß (41%±2.63%). However, no reduction in luciferase activity was observed in the cells transfected with Luc-C/EBPß-M, Luc-Gata2-M, and Luc-Foxg1-M (Fig. 8B). It suggests that the C/EBPß, Gata2 and Foxg1 receptors are direct targets of miR-92b in fetal liver cells. we next constructed target gene expression vectors with complete 3′UTR or mutant 3′UTR to identify the major functional target gene involved in differentiation arrest by miR-92b. These expression vectors were stablely transfected into the miR-92b overexpressed EpCAM^+^ fetal liver cells 7 days after induction to differentiate *in vivo*. At 48 hours after transfection, the ALB expression levels in these transfected cells were measured using Western blot. ALB expression level in the cells transfected with C/EBPß-M had significantly increased compared to that in control EpCAM^+^ fetal liver cells transfected with blank expression vector. While ALB expression levels were not increased in the cells transfected with C/EBPß, Foxg1, Foxg1-M, Gata2 and Gata2-M, while the western analysis of AFP showed that AFP expression was contrarily to ALB expression (Fig. 8C). Cell cycle analysis showed the rescue effects of C/EBPß in miR92b overexpressed fetal liver cells. The significantly increase in G1 phase and decrease in S and G2/M phase could be observed when restoring the C/EBPß expression in miR-92b overexpressed fetal liver cells by transfecting pcDNA-C/EBPß-M (Fig. 8D). It suggests that restoration of C/EBPß gene expression in miR-92b overexpressed fetal liver cells reversed the differentiation arrest by miR-92b. In addition, we confirmed that results that the C/EBPß is direct targets of miR-92b by western bolt (Fig. S2).

## Discussion

Increasing evidence suggest the presence of LCSCs in many HCC cell lines and primary HCC tissue. However, the mechanism of neoplastic transformation of LCSCs has not yet been studied. We tried to explore the mechanism of transformation in a rat hepatocarcinogenesis model. A major advantage of this model is that the normal hepatic stem cells and LCSCs with the same marker and genetic background were simultaneously enriched. This facilitates the studies on different genetic factors with regard to neoplastic transformation of LCSCs. In this study, a comprehensive analysis of differential expression of miRNAs and their important targets were performed between EpCAM^+^ fetal liver cells and EpCAM^+^ liver cancer cells induced by DEN. The strong link on a cellular origin between them has been proven in the chemical hepatocarcinogenesis model [23], [24]. Our result revealed that significant dysregulation of some specific miRNAs found in LCSCs, and they maybe play critical roles as an oncogen or tumor suppressor gene in hepatic stem cells during the neoplastic transformation. These altered miRNAs were almost identical to those observed in primary human HCCs. miR-21, miR-10b and miR-34c have been reported to be upregulated in various types of cancers, including HCC [25]. Notably, some of these miRNAs previously have been found to be dramatically altered during the hepatocarcinogenesis induced by the chemical carcinogen. For example, the expression of tumor suppressor genes, like PTEN [26] and P53 [27], which are the targets of miR-21 and miR-34c, were significantly reduced in the premalignant foci and HCC nodules [28]. Apart from the role of oncogen, some of these dysregulated miRNAs also regulates cancer stem cells. The miRNA family, let-7i and let-7 have been found to regulate the key features of breast cancer stem cells like a self-renewal, multipotent differentiation and tumorigenicity [29]. The miR-200a was observed regulates epithelio-mesenchymal to stem-like transition in nasopharyngeal carcinoma cells by targeting ZEB2 and ß-catenin signaling genes [30]. However, the role of these specific miRNAs and its target genes in neoplastic transformation of LCSCs remain to be elucidated.

The cancer stem cell model is consistent with the idea that oncogenic mutations often act by causing differentiation arrest [31]. Indeed, the differentiation arrest is a prominent feature of cancer stem cells that distinguish them from normal stem cells besides tumorigenecity. This phenomenon usually observed in the rat hepatocarcinogenesis model [32]. As we know, one of the most vital biological functions of miRNA is to regulate the gene expression that controls the biological process in development and differentiation of stem cells. Thus, it is reasonable to speculate the probable role of miRNAs in differentiation arrest of LCSCs.

The first important finding our current study is the functional role of miR-92b involved in the differentiation of hepatic progenitors. The significant maturation arrest was found in EpCAM^+^ cells isolated from the miR-92b overexpressed fetal liver cells detected by both *in vitro* and *in vivo* differentiation assay. To identify the probable targets involved in differentiation arrested by miR-92b, we performed target prediction based on miRanda algorithm and predicted target cDNA microarray. Final analysis of gene function classification pinpointed three transcription factor genes are directly involved in the differentiation regulated by miR-92b. These transcription factors are functioning as anexecutors of several important growth factors signaling pathway, which regulate differentiation and pluripotency in stem cells. C/EBPß is one of verified targets by using 3′UTR reporter assay, which belongs to a class of DNA binding proteins named bZIP proteins. The bZIP transcription proteins play a very important role in balancing the cellular differentiation and proliferation, C/EBPß proteins are crucial regulators of the balance between differentiation and proliferation in various tissues [33]. It has been inferred that during hematopoiesis, the C/EBPß regulatory pathway is helpful for normal cell differentiation and development [34]. It has been reported that ectopic expression or inducible activation of C/EBPß promotes morphologic differentiation. Although enhanced activity of C/EBPß can promote differentiation of normal and malignant myeloid precursors, a complete loss of C/EBPß activity might be necessary to disrupt the differentiation potential of CML-BC (chronic myelogenous leukemia blast crisis) progenitors [35]. Using the rat pancreatic cell line AR42J-B13, researchers showed that transfection with C/EBP-βprovoked hepatic differentiation [36], while transfection with a dominant negative inhibitor of C/EBP-β will both inhibit glucocorticoid-induced formation of hepatocytes and also caused loss of the hepatic phenotype from cells maintained for a long periods in dexamethasone [37]–[39]. C/EBPß is also activated in organ cultures of embryonic pancreas following glucocorticoid treatment and its expression is associated with hepatic differentiation [37], [40]. Our results indicated that miR-92b played the role most probably by influencing C/EBPß. C/EBPß regulates an early liver development of mouse embryo [36]. During hepatic differentiation of hepatic progenitors, C/EBPß directly stimulates the transcription of many liver-specific genes (ALB, glucose-6-phosphatase and tyrosine aminotransferase) after be phosphorylated in its activation domain which activated by some growth factor signal pathways including, TGF-ß, EGF, etc [41]. AFP is one of the classic liver cancer indicators, which positively correlates to the differentiation degree of malignancy of cancer. Thus, AFP is positively related to the cell immature level. Because EpCAM is a stem-cell like symbol and reasonably AFP level is already higher in the EpCAM^+^ cells, the change of AFP mRNA level may be not as obvious as ALB. The essential role of C/EBPß in hepatocyte maturation is also verified by Greenbaum et al [42]. This study reporting for biological consequence of C/EBPß gene knockout suggests that knock-out mice lacking an expression of C/EBPß have impaired responses to partial liver hepatectomy with hepatocytes generally showing reduced expression of immediate-early response genes. This phenomenon is similar with our result of *in vivo* differentiation assay, which indicates that C/EBPß may be a critical downstream target of miR-92b. Although the gene sequences of human and mouse are not absolutely the same, our experiment can be used as a reference. And we can accordingly predict that C/EBPß might also be one of the key target genes of the miR92b in human species, which needs further study.

The proliferation of hepatic progenitors is strictly regulated during liver development. Hepatic progenitors are normally quiescent in the adult liver, but they can be induced for proliferation in response to injuries or cytokine stimulation. The balance between proliferation and differentiation usually is broken during tumorigenesis. The current study addition to differentiation arrest, proliferation was significantly increased in the miR-92b overexpressed EpCAM^+^ fetal liver cells. We uniquely demonstrated that percentage of miR-92b overexpressed group in S and G2/M phase significantly increased compared with control group. In addition to its role as transactivator of many genes, C/EBPß is also be found to arrest the progression of the cell cycle from G1 to S-phase in hepatoma cells and mediates the proliferative effects of TGF in primary mouse hepatocytes [43]. Recently, Sengupta et al [44] found that overexpression of miR-92b in human embryonic stem cells would prompt cells progression from G1 phase into S phase by targeting a G1/S checkpoint gene p57, which is not consistent with our results. Whether p57 gene also involved in the changes of cell cycle caused by overexpression of miR-92b in the hepatic progenitors needs further investigation.

Our data showed that the mir-92b might contribute to the malignant transformation of the liver stem cells, which may be a new valuable marker of HCC, and the analogy between the already confirmed miRNAs such as antioncogene mir-200a [45] or Oncogene mir-21 [46]. miRNA-200a and miR-21 were found to be involved in the malignant transformation of liver stem cells. However, the transformation into “real liver cancer stem cells” by these miRNAs has not been achieved yet. There are two ways for solving this problem: finding new miRNA or combining the investigations of multiple miRNAs. Our investigation is based on the first consideration, which was supported by that miR-92b may also play an important role in the malignant transformation besides to miR-200a and miR-21. In addition, this study may also provide new members for the combined study of the multiple miRNAs, which were much worth to be done. In our later study, we are planning to combine the miR-92b with miR-200a or miR-21 to search for the mechanisms of malignant transformation.

In conclusion, we identified miRNAs that are aberrantly expressed in EpCAM^+^ liver cancer cells. The overexpressed miR-92b promoted EpCAM^+^ fetal liver cells proliferation and arrested their differentiation by targeting C/EBPß genes. The EpCAM(+) cells will most probably presented as immature cells, the mir-92b is a inhibitor of cell differentiation, so it is reasonable that the expression level of mir-92b is higher in EpCAM(+) LCSCs. Buechner J. et al revealed that mir-92b expression was downregulated in MYCN ((v-myc myelocytomatosis viral related oncogene, neuroblastoma derived (avian)) repressed SK-N-BE cells [47], In addition, MYCN-regulated mir-92b that could target the 3^,^ UTR sequence of DKK3 (Dickkopf-3), which functions as a tumor suppressor in a range of tumors [48]–[50]. However, further studies involving analysis of mechanism of miR-92b upregulation in liver cancer stem cells are required. These findings have implications for describing new mechanisms for miRNA mediated regulation of hepatocarcinogenesis.

## Supporting Information

Figure S1 AFP expression in EpCAM^+^ liver cancer cells (a), EpCAM^+^ fetal liver cells (b), EpCAM^−^ liver cancer cells (c) and EpCAM^−^ fetal liver cells (d).
(TIF)

Figure S2 C/EBPβ expression level in the miR-92b overexpressed EpCAM^+^ cells had significantly decreased compared to that in control cells.
(TIF)
