# Supplementary figures and images for: Upregulated MicroRNA-92b Regulates the Differentiation and Proliferation of EpCAM-Positive Fetal Liver Cells by Targeting C/EBPß
Source: PLoS One. 2013 Aug 2;8(8):e68004. doi: 10.1371/journal.pone.0068004 (PMC3732262; doi:10.1371/journal.pone.0068004)

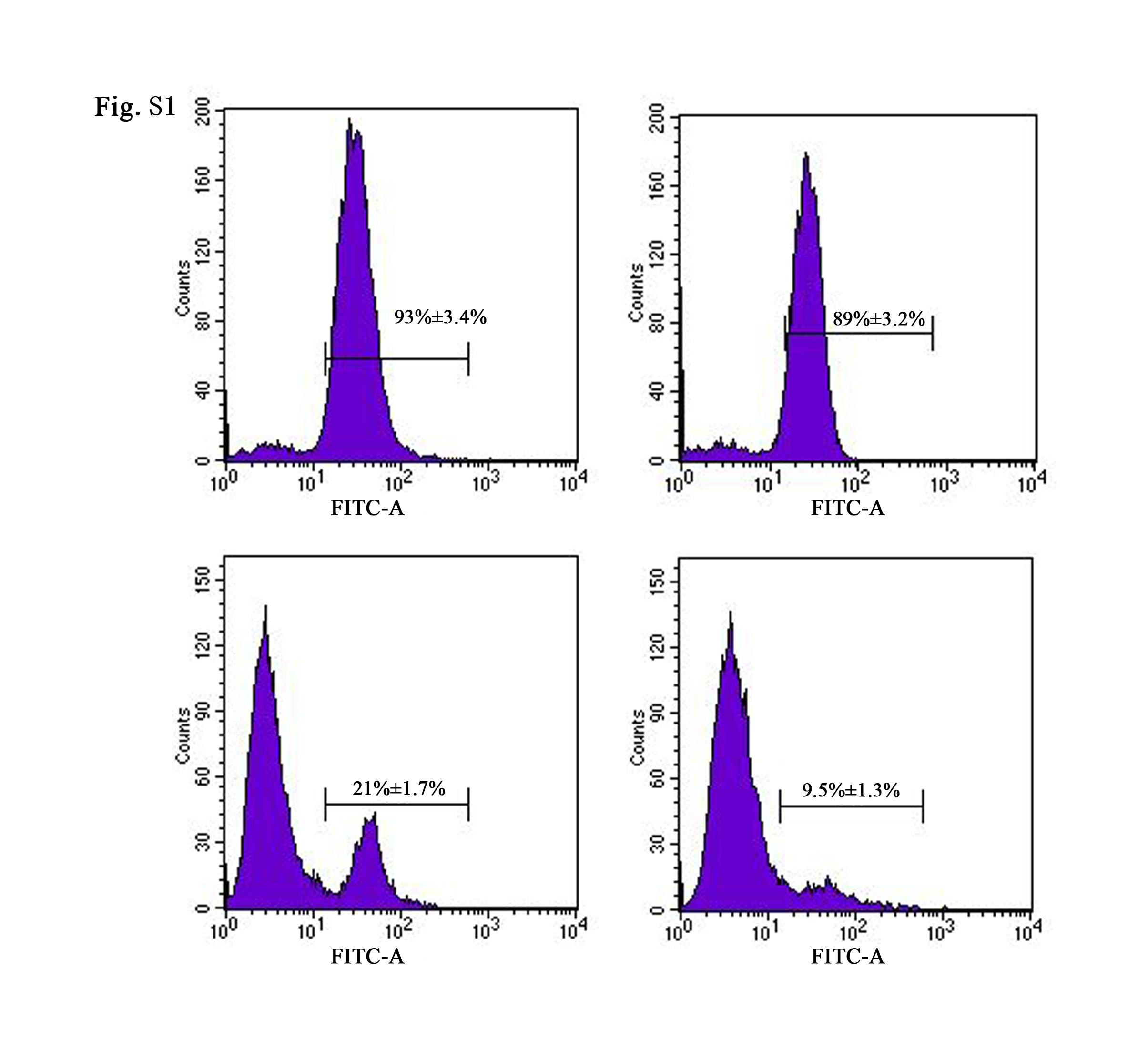

Supplement: Figure S1 — AFP expression in EpCAM+ liver cancer cells (a), EpCAM+ fetal liver cells (b), EpCAM− liver cancer cells (c) and EpCAM− fetal liver cells (d). (TIF) [file pone.0068004.s001.tif]

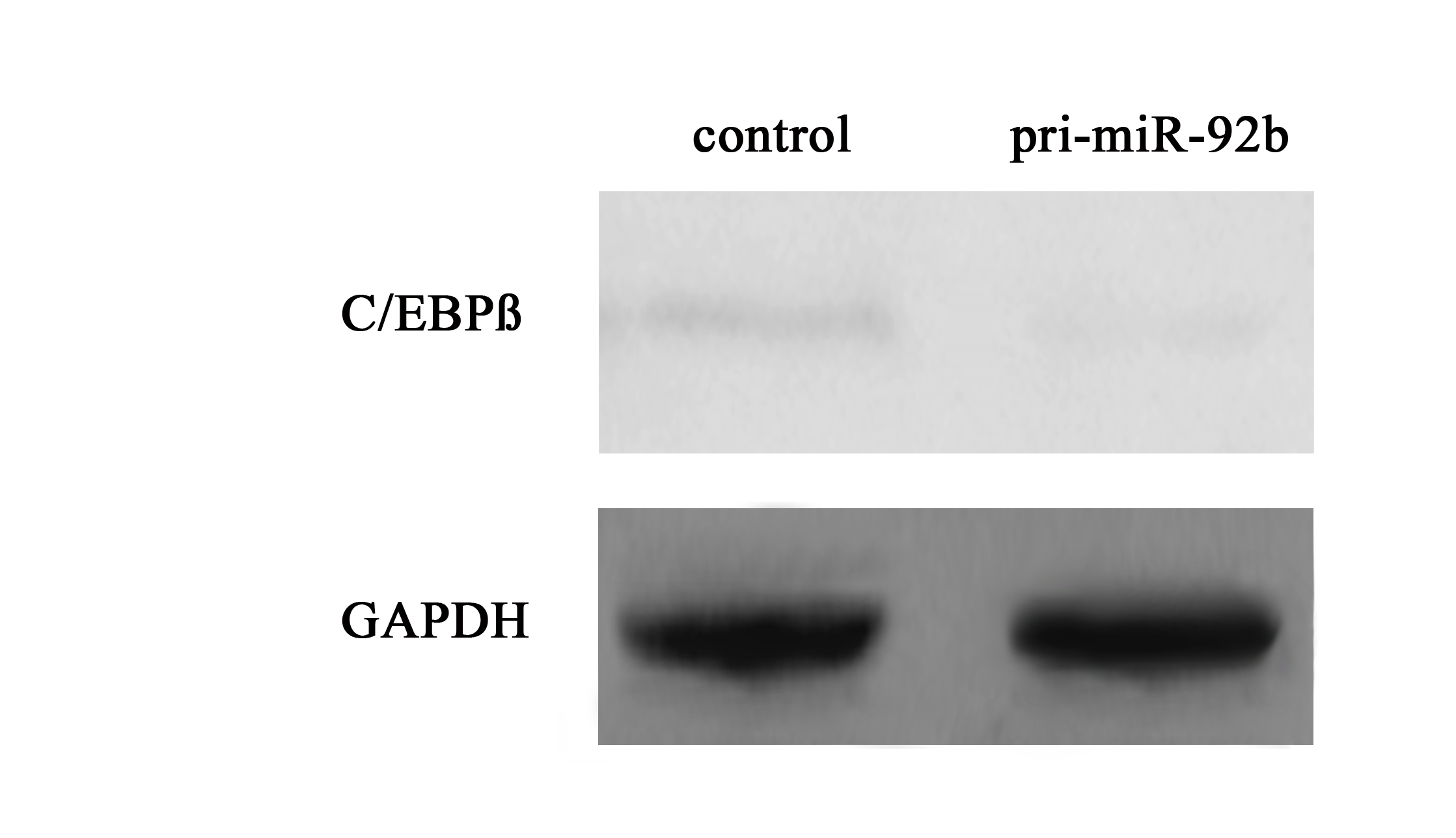

Supplement: Figure S2 — C/EBPβ expression level in the miR-92b overexpressed EpCAM+ cells had significantly decreased compared to that in control cells. (TIF) [file pone.0068004.s002.tif]
